# Cold-Pressed *Aristotelia chilensis* (Mol.) Stuntz Seed Oil Prevents Metabolic-Dysfunction-Associated Steatotic Liver Disease (MASLD) in a High-Fat-Diet-Induced Obesity Murine Model

**DOI:** 10.3390/antiox13111384

**Published:** 2024-11-13

**Authors:** Benjamín Claria, Alejandra Espinosa, Alicia Rodríguez, Gretel Dovale-Rosabal, José Luis Bucarey, María Elsa Pando, Nalda Romero, Francisca Reinoso, Camila Sánchez, Rodrigo Valenzuela, Carolina H. Ribeiro, Santiago P. Aubourg

**Affiliations:** 1 Department of Food Science and Chemical Technology, Faculty of Chemical and Pharmaceutical Sciences, Dr. Carlos Lorca Tobar 964, University of Chile, Santiago 8380494, Chile; benjamin.claria@ug.uchile.cl (B.C.); gretel.dovale@ug.uchile.cl (G.D.-R.); nromero@uchile.cl (N.R.); francisca.reinoso@ug.uchile.cl (F.R.); camila.sanchez.s@ug.uchile.cl (C.S.); 2 Department of Medical Technology, Faculty of Medicine, University of Chile, Santiago 8380000, Chile; ealejand@uchile.cl; 3 School of Medicine, Faculty of Medicine, Universidad de Valparaíso, San Felipe 2172972, Chile; jose.bucarey@uv.cl; 4 Center of Interdisciplinary Biomedical and Engineering Research for Health, Universidad de Valparaíso, San Felipe 2172972, Chile; 5 Department of Nutrition, Faculty of Medicine, University of Chile, Santiago 8380000, Chile; pandosanmartin@uchile.cl (M.E.P.); rvalenzuelab@uchile.cl (R.V.); 6 Immunology Program, Biomedical Sciences Institute (ICBM), Faculty of Medicine, University of Chile, Santiago 8380000, Chile; chager@uchile.cl; 7 Department of Food Technology, Marine Research Institute (CSIC), Eduardo Cabello 6, 36208 Vigo, Spain

**Keywords:** cold-pressed maqui seed oil, α-, β-, γ-, and δ-tocopherol, α-tocotrienol, metabolic fatty liver disease, experimental diet, biochemical markers, HFD male mice model, obese-related biological parameters

## Abstract

This study evaluated the effects of cold-pressed maqui (*Aristotelia chilensis* (Mol.) Stuntz) seed oil (MO) on liver metabolism and biochemical markers in a high-fat diet (HFD) murine model. In it, the fatty acid profile, tocopherol and tocotrienol contents, and antioxidant capacity of MO were analyzed. Male C57BL/6 mice were divided into four groups (i.e., a, b, c, and d groups) and supplemented for 12 weeks according to the following distribution: (a) control diet (CD)-sunflower oil (SO), (b) CD+MO, (c) HFD+SO, and (d) HFD+MO. Total body and organ weights, serum markers, and liver fat infiltration were assessed. MO contained 32.31% oleic acid, 46.41% linoleic acid, and 10.83% α-linolenic acid; additionally, α- and γ-tocopherol levels were 339.09 ± 5.15 and 135.52 ± 38.03 mg/kg, respectively, while β-, δ-tocopherol, and α-tocotrienol were present in trace amounts and the antioxidant capacity measured was 6.66 ± 0.19 μmol Trolox equivalent/g. MO supplementation significantly reduced the visceral fat (0.76 ± 0.06 g vs. 1.32 ± 0.04 g) and GPT (glutamate pyruvate transaminase) levels (71.8 ± 5.0 vs. 35.2 ± 2.6 U/L), and the liver fat infiltration score (6 vs. 3) in the HFD+MO group compared to HFD+SO. It is suggested that MO may effectively prevent fatty liver disease, warranting further research on its potential benefits for human health.

## 1. Introduction

Non-alcoholic fatty liver disease (NAFLD) has emerged as a global health concern, encompassing a spectrum of liver conditions ranging from simple steatosis to non-alcoholic steatohepatitis (NASH) [1,2]. In recent years, a paradigm shift has led to the renaming of NAFLD as metabolic-dysfunction-associated steatotic liver disease (MASLD) to better reflect its multifaceted etiology and clinical implications [3,4]. Accordingly, MASLD is accompanied by metabolic syndrome and obesity, which are significantly associated with cardiometabolic risk, thus including insulin resistance, hypertension, or dyslipidemia [3]. One critical aspect of MASLD progression is the accumulation of intrahepatic fat, due to an imbalance of fatty acid input and output, a key factor both in the onset of hepatic steatosis (HE) and its progression towards metabolic steatohepatitis [5]. Oxidative stress is one of the key mediators of hepatic damage and is a major contributor to the progression from simple steatosis to steatohepatitis [6].

The beneficial potential effect of antioxidant-rich nutraceuticals on MASLD has been described, suggesting that several classes of antioxidants may be considered in potential treatments for hepatic steatosis, likely through an indirect effect on mitochondrial function [7,8]. *Aristotelia chilensis* (Mol.) Stuntz, commonly known as maqui, is an endemic Chilean tree whose fruit is rich in polyphenols and other bioactive compounds that show strong antioxidant activity [9].

Nevertheless, maqui seed oil extracts have been explored for their antioxidative and enzyme-inhibitory properties only in recent years [10]. Maqui seed oil contains high levels of β-sitosterol, as well as tocopherols and tocotrienols [10], which can be divided into eight different isoforms: α-, β-, γ-, and δ-tocopherols, and α-, β-, γ-, and δ-tocotrienols [11]. α-Tocopherol has been regarded as the main isoform of vitamin E, specifically for its bioavailability, antioxidant behavior, and modulating various signaling pathways along with other vitamin E isoforms [11]. However, composition of fruit seed oils can vary due to differences in the kind of extraction, which may influence the nutraceutical properties [12]. Therefore, a better characterization of cold-pressed maqui seed oil (MO) may lead to greater health benefits and prevention of liver steatosis and other metabolic parameters associated with MASLD. The current work aimed to explore the potential benefit of MO supplementation for obesity-associated metabolic alterations in mice receiving a high-fat diet regimen.

## 2. Materials and Methods

### 2.1. Raw Seed Oil, Chemical Reagents, and Standards

The raw material “cold-pressed maqui seed oil” (*Aristotelia chilensis* (Mol.) Stuntz) (MO) was purchased from De Castañas y Amores SpA (Santiago, Chile); it was preserved at −80 °C and protected from light and humidity in PET (polyethylene terephthalate) bottles. Methyl tricosanoate internal standard and gas–liquid chromatography (GLC) reference standard GLC-463 were obtained from Nu-Chek-Prep, Inc. (Elysian, MN, USA). Hydrogen, nitrogen, and zero-air gas cylinders were purchased from GasLab-Linde (Santiago, Chile). Trolox (6-hydroxy-2,5,7,8-tetramethylchroman(E)-2-carboxylic acid), AAPH (2,2′-Azobis(2-methylpropionamidine)dihydrochloride), fluorescein, α-, β-, γ- and δ-tocopherol, α-tocotrienol and gallic acid standards, and other chemical reagents were purchased from CalbioChem Merck (Santiago, Chile).

### 2.2. Identification and Quantification of Fatty Acids (FAs) Using GLC

The composition of FAs in MO was carried out by conversion to FA methyl esters (FAMEs) through alkaline and acid methylation, according to the method described by IUPAC [13]. In order to determine and quantify FAs, the procedure was performed according to AOCS Ce 1j-7 [14]. The FA profile and quantification were determined using the GLC Shimadzu gas chromatograph (Kyoto, Japan) equipped with a flame ionization detector, split injection system, and a capillary column (100 m × 0.25 mm i.d. × 0.2 µm) SPTM-2560 (Supelco, Bellefonte, PA, USA). The oil was kept in the oven for three minutes at 160 °C, followed by an increase of 1 °C·min^−1^ until reaching 230 °C. Both the injector and flame ionization detector temperatures were 240 °C. The carrier gas was hydrogen. For the qualitative determination, the retention times of the sample were compared to the retention times of a previously injected standard. FAMEs were identified by comparison to the reference standard GLC-463 (Nu-Chek Prep, Elysian, MN, USA). The quantification of all individual FAs (g·100 g^−1^ total FAs, TFAs) was achieved using methyl tricosanoate (C23:0 methyl ester) as an internal standard according to the AOCS method [14].

### 2.3. Identification and Quantification of Tocopherols and Tocotrienols

Tocopherol and tocotrienol contents were determined by high-performance liquid chromatography (HPLC) according to the AOCS standard method [15], using an HPLC consisting of a Merck–Hitachi pump L-6200A (Merck, Darmstadt, Germany), a Rheodyne 7725i injector with 20 μL sample loop, a LiChroCART Superspher Si 60 column (25 cm × 4 mm id, 5 μm particle size; Merck, Darmstadt, Germany), a Hitachi Chromaster 5440 fluorescence detector, and a PC with Clarity chromatography software in order to process the chromatographic signals. First, 100 mg of the sample were added to a 10 mL volumetric flask. The volume was then completed with HPLC-grade hexane and stirred to dissolve the sample. For the HPLC determination, 80 µL of the tocopherol and tocotrienol standard solutions, with a concentration of 3 µg·mL^−1^, were injected in the chromatographic system, and the corresponding peak areas of tocopherol and tocotrienols were recorded. Subsequently, 80 µL of the sample solution and its duplicate were injected. Tocopherols and tocotrienols were identified and quantified based on their chromatograms. The mobile phase was propan-2-ol in hexane (0.5/99.5, *v*/*v*) at a flow rate of 1 mL·min^−1^. Chromatographic peak signals were detected at 290 nm (excitation) and 330 nm (emission). Tocopherol and tocotrienol standards used were obtained from Merck (Darmstadt, Germany). Results were expressed as mg tocopherols or tocotrienols·kg^−1^ oil according to the following equation:

(1) Tocopherol or tocotrienol concentration=C×a×V×(A×m)⁻¹(mg·kg⁻¹ oil)

*C:* Standard concentration (µg·mL^−1^)

*A*: Standard area (mVs)

*a*: Sample area (mVs)

*m*: Sample mass (g)

*V*: Volumetric flask volume (mL)

### 2.4. Antioxidant Capacity Determination (H-ORAC_FL_)

The antioxidant capacity was determined using the hydrophilic oxygen radical absorbance capacity (H-ORAC_FL_) assay according to the procedure described by Prior et al. [16] and modified by Fuentes et al. [17].

### 2.5. Mice Protocol

Twenty-four male C57BL/6 mice were obtained from the Public Health Institute of Chile. The animals were kept in the Animal Maintenance Unit (UMT) of the Nutrition Department of the Faculty of Medicine, University of Chile, in a room with a constant temperature (21–23 °C) and with light and dark cycles of 12 h each. After two weeks of acclimatization, the mice were randomly divided into four groups (*n* = 6 for each group) and fed with the specific pellet described in the following section. All the procedures performed in this study were approved by the Institutional Animal Care and Use Committee (CICUA) of the University of Chile (Protocol no. 22567-MED-UCH). The four mice groups were fed as follows: control diet (CD) plus sunflower oil (SO) or MO and high-fat diet (HFD) plus SO or MO. Treatments were administered for 12 weeks. The total calories of the HFD were 5.2 kcal·g^−1^, with 60% fat, 20% protein, and 20% carbohydrates (D12492, Research Diets, New Brunswick, NJ, USA). The control mice were given a CD (D12450J, Research Diets, New Brunswick, NJ, USA) consisting of 3.8 kcal·g^−1^, 10% fat, 20% protein, and 70% carbohydrates. After 11 weeks, a glucose tolerance test was performed. After a 4 h fast at the end of the 12th week, mice were weighed and then euthanized. Liver, visceral fat, and epididymal fat were subsequently extracted, weighed on an analytical scale (Radwag AS60/220.r2), and stored at −80 °C until further analysis.

### 2.6. Measurements of Serum Parameters

After blood was collected through cardiac puncture, the serum was separated by centrifugation at 3000× *g* for 15 min at room temperature. Glutamate pyruvate transaminase (GPT), glutamate oxaloacetate transaminase (GOT), triacylglycerols (TGs), total cholesterol (T-Chol) and HDL-cholesterol (HDL-Chol) were assayed using dry chemistry technology (SPOTCHEM EZ, Minneapolis, MN, USA). Serum insulin concentrations were determined by ultrasensitive mouse immunoassay (Mercodia, Uppsala, Sweden). The HOMA-IR (homeostasis model assessment of insulin resistance) was calculated as follows: HOMA-IR = [fasting glucose (mg·dL^−1^) × fasting insulin (µU·mL^−1^)]/405.

### 2.7. Histological Assessment

Liver samples underwent dehydration, bleaching, and paraffin embedding for histological analysis. Microscopic slides with 5 µm cuts were stained with Mayer’s hematoxylin and 1% aqueous eosin to visualize nuclei in blue and cytoplasm in pink. Histological preparations were examined by a bright field microscope (Leica DM500, Wetzlar, Germany), and 20–25 images were obtained for each preparation. Images were analyzed with ImageJ software 1.8.0 (NIH, Bethesda, MD, USA) to evaluate steatosis percentage, opening the image and converting it into an 8-bit image. Then, the threshold was set to highlight the areas of steatosis by adjusting the threshold levels. The area of steatosis was measured using the area fraction in each image. The score of each mouse was determined according to the scoring system for rodents [18].

### 2.8. Bodipy Stain in Liver Cryosections

Immediately after euthanasia, liver tissues were excised, washed in cold phosphate-buffered saline (PBS) to remove blood, and flash-frozen in liquid nitrogen. Samples were stored at −80 °C until further processing. Frozen liver tissues were embedded in an optimal cutting temperature (OCT) compound and sectioned at 10–12 μm thickness using cryostat equipment (Model CM1850, Leica Biosystems, Deer Park, IL, USA). Sections were collected on poly-L-lysine-coated slides to enhance tissue adhesion and stored at −20 °C until staining. After fixation (4% paraformaldehyde for 10 min), sections were washed three times with PBS for 5 min each. The sections were further incubated with Bodipy 493/503 and DAPI stain (1 µg·mL^−1^ in PBS) (Thermo Fisher Scientific, Waltham, MA, USA) in a dark and humidified chamber for 30 min at room temperature. After incubation, slides were washed three times in PBS to remove excess dye. Green and blue fluorescence signals were captured by confocal microscopy (Nikon Spectral C2 + microscope, Tokyo, Japan).

### 2.9. Statistics

Data obtained are presented as the mean ± standard error of the mean (SEM). Significant differences between and within multiple groups were examined using an ordinary two-way ANOVA test, followed by Tukey’s post hoc test for multiple comparisons; * *p* < 0.05 (95% CI) was considered statistically significant. Prior to the ANOVA test, data were checked for normality using the Shapiro–Wilk and Kolmogorov–Smirnov tests, and for homoscedasticity using the Spearman’s rank correlation test to ensure that the assumptions of the ANOVA were met. All statistical analyses were performed using GraphPad Prism 10.3.0 (San Diego, CA, USA).

## 3. Results

### 3.1. MO Characterization

The FA composition and quantification of MO are shown in Table 1 where, 12 peaks of FAs were detected, among which linoleic acid (C18:2 9c 12c), oleic acid (C18:1 9c), and α-linolenic acid (C 18:3 9c 12c 15c) were the most abundant, representing 89.5% of the total FAs.

Table 2 shows a remarkable presence of α-tocopherol and γ-tocopherol compounds in MO, these showing values of 339.09 ± 5.15 and 135.52 ± 3.50 mg·kg^−1^ oil, respectively. Meanwhile, β-, and δ-tocopherol and α-tocotrienol were detected in trace values. Bastías-Montes et al. [10] reported lower values for tocopherol compounds than in the current study; even though these authors also analyzed MO, such tocopherol content variability may be explained on the basis of differences in the kind of oil extraction.

The antioxidant capacity value measured by hydrophilic oxygen radical absorbance capacity (H-ORAC_FL_) assay of MO is shown in Table 3.

### 3.2. MO Effects on Tissue Weight and Biochemical Parameters of Obese Mice

**Figure 1:**
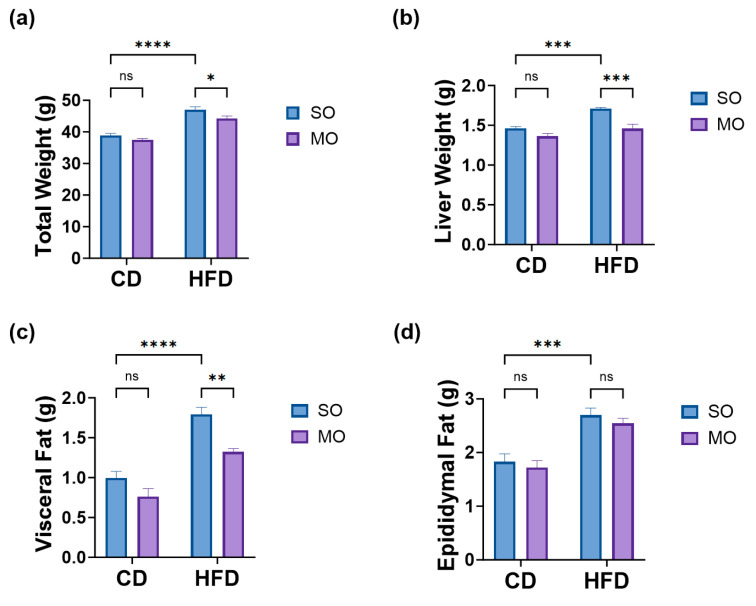
Effect of cold-pressed maqui seed oil (MO) on total and adipose tissue weight of mice under an HFD. Animals were weighed before euthanasia (**a**), followed by an immediate measure of the liver (**b**), visceral fat (**c**), and epididymal fat (**d**) weight. Abbreviations: CD (control diet), HFD (high-fat diet), and SO (sunflower oil). Data are presented as the mean ± standard error of the mean (SEM). Statistical differences were determined using ordinary two-way ANOVA test, followed by Tukey’s comparative test: * *p* < 0.05; ** *p* < 0.01; *** *p* < 0.001; **** *p* < 0.0001; ns: no significant differences; *n* = 6 mice for each group.

In order to assess the potential effect of MO supplementation on obesity-related biological parameters, four groups of six C57BL/6 mice each were included in this study. Figure 1 provides the comparative results on: (a) total weight, (b) liver weight, (c) visceral fat, and (d) epididymal fat of mice fed with CD supplemented with SO (CD+SO), CD supplemented with MO (CD+MO), HFD supplemented with SO (HFD+SO), and HFD supplemented with MO (HFD+MO).

As expected, mice fed with HFD+SO displayed higher weight compared to mice that received CD+SO diet (Figure 1, panels a–d). Supplementation of HFD+MO significantly prevented HFD-associated increase of total mice weight (*p* < 0.05), liver (*p* < 0.001), and visceral (*p* < 0.01) weight (Figure 1, panels a–c), although this regimen had no significant effect on epididymal weight (*p* > 0.05) (Figure 1d). No difference in total and adipose tissue weight was observed in control groups (CD+SO and CD+MO) (*p* > 0.05).

As shown in Table 4, the biochemical profile was measured in the serum of mice from the four groups. An intraperitoneal glucose tolerance test (iGTT) was performed before euthanasia of mice receiving CD or HFD dietary regimens with oil supplementation. Glucose content increased significantly in the group of mice fed with HFD+SO (*p* < 0.05) when compared to the mice fed with CD+SO, as well as in the group of mice fed with HFD+MO (*p* < 0.05) when compared to the mice fed with CD+MO. No statistical differences (*p* > 0.05) were found among the four groups in iGTT (represented by the area under the curve, AUC), fasting insulin serum concentration, and HOMA-IR. Hepatic glutamate pyruvate transaminase (GPT) activity significantly decreased in mice fed with CD+MO (*p* < 0.05) and HFD+MO (*p* < 0.01) diets compared to mice fed with CD+SO and HFD+SO, while hepatic glutamate oxaloacetate transaminase (GOT) activity was not different among groups. In addition, no difference in TG serum concentration was detected between CD+SO- and HFD+SO-fed mice, although supplementation of CD+MO and HFD+MO increased the TG levels in both groups (*p* > 0.05). No significant changes (*p* > 0.05) were observed in the total cholesterol (T-Chol) and HDL-cholesterol (HDL-Chol) values between HFD+SO and HFD+MO groups.

### 3.3. Effect of Maqui Seed Oil on MASLD-Associated Liver Damage

**Figure 2:**
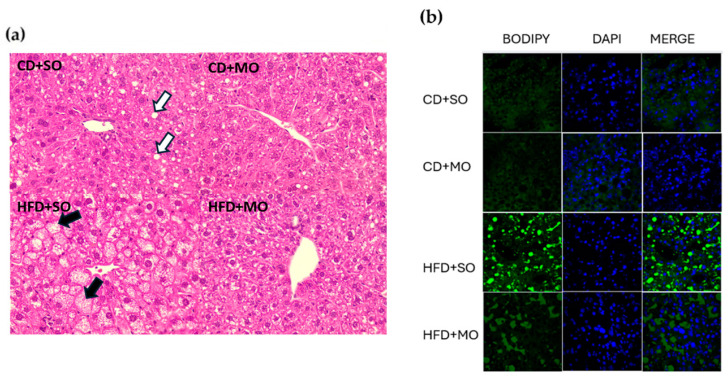
Histology of liver steatosis prevention by cold-pressed maqui seed oil supplementation. (**a**) Hematoxylin and eosin (H&E) staining of paraffin liver sections displaying general architecture and cellular morphology. White arrows indicate macrovesicular steatosis and black arrows microvesicular steatosis. (**b**) Bodipy staining on a representative liver cryosection for lipid detection (green fluorescence) merged with DAPI stain (blue). Abbreviations: CD (control diet), HFD (high-fat diet), SO (sunflower oil), and MO (cold-pressed maqui seed oil); *n* = 6 mice for each group. Magnification of the figure is 400×.

Histological evaluation has reported that MASLD is associated with fat accumulation of liver for values higher than 5% in hepatocytes [14]. Figure 2 shows fatty liver infiltration through histological analysis of liver samples from mice receiving CD or HFD with oil supplementation (SO or MO). Figure 2a shows the most representative liver section micrograph staining with hematoxylin (cell nucleus) and eosin (cytoplasm) (H&E) for each treatment group. White arrows denote the presence of macrovesicular steatosis (large lipid droplets in hepatocytes), and black arrows mark microvesicular steatosis (small lipid droplets in hepatocytes). The magnification of the figure is 400×. In Table 5, the steatosis score (macrovesicular steatosis plus microvesicular steatosis plus hypertrophy and inflammation with a maximum score of 12) is calculated to a murine model according to Liang et al. [18]. Thus, macrovesicular steatosis and microvesicular steatosis were both separately scored; based on the percentage of the total area affected, the severity was graded into the following category groups: 0 (<5%), 1 (5–33%), 2 (34–66%), and 3 (>66%). The difference between macrovesicular and microvesicular steatosis was defined on the basis of whether the vacuoles displaced the nucleus to the side (macrovesicular) or not (microvesicular).

According to the present study, 50% of HFD+SO-fed mice developed steatosis after 12 weeks of nutritional regimens. In Figure 2a, black arrows revealed microvesicular steatosis present in the HFD+SO group, reaching a mice score of 6/12 versus 1/12 from the CD+SO-fed mice (Table 5). Although Figure 2a shows macrovesicular steatosis (white arrows) in the CD+SO group, Table 5 indicates that the steatosis score of mice with CD+SO is 1/12, thus indicating no steatosis. MO administration did not affect the steatosis score in CD+MO-fed mice; however, mice on HFD+MO showed a decreased score for micro- and macrovesicular steatosis (score of 3/12). No inflammatory focus was observed in the four groups of animals (Table 5). Figure 2b shows, in fluorescent green, the lipid droplets’ distribution in the liver tissue of the four mice groups. As in Figure 2a, the HFD+SO mice group exhibits steatosis, and in the HDF+MO mice group, the prevention effect of MO supplementation is observed due to the fewer green lipid droplets detected. Therefore, these results suggest a potential beneficial effect of MO on hepatic steatosis in obese mice fed with HFD.

## 4. Discussion

In the current work, MO was characterized and tested in C57BL/6 mice to explore its potential benefits against MASLD. The composition of FAs present in MO shows a complex variety of monounsaturated and polyunsaturated FAs, with the second group showing a higher presence than the first one; remarkably, both FA groups constitute 90.6% of TFAs. These results are similar to those obtained by Sánchez et al. (2024) in the extraction of freeze-dried maqui oil [19], and Bastías-Montes et al. [10] in pressed maqui seed oil, where the polyunsaturated FAs were more abundant than monounsaturated FAs and the content of both FA groups was 88.5% and 88.1%, respectively.

Generally, beneficial components in vegetable oils are alpha-linolenic acid (ALA), a precursor of long-chain polyunsaturated FA n-3, and its antioxidant-derived molecules [20]. The current content of ALA was only 10.83%, a minor percentage compared to rosa mosqueta oil (33%) or chia oil (64%) [21]. On the other hand, the presence of high levels of α- and γ-tocopherol in MO can be considered of great interest. Tocopherol compounds, well known for their antioxidant properties, can significantly reduce oxidative stress, ferroptosis, and lipid peroxidation [22,23], which are often elevated in the livers of patients with metabolic dysfunction [24]. It is worth pointing out that the content of α- and γ-tocopherol in MO (339.09 ± 5.15 and 135.52 ± 3.50 mg·kg^−1^ oil, respectively) is higher than twice the amount of these compounds found in chia seed oil [25] or extra virgin olive oil [12] and over 15 times the amount in grapeseed oil [26]. Hendawy et al. [27] found that the content of α- and γ-tocopherol of cold-pressed raspberry seed oil was 42.73 and 134.62 mg·100 g^−1^ oil, respectively, with a total concentration of 185.1 mg tocopherols·100 g^−1^ oil. Therefore, MO may be considered an important source of tocopherols that could reduce MASLD steatosis [27].

The hydrophilic oxygen radical absorbance capacity measured with fluorescein (H-ORAC_FL_) assay showed that MO has an antioxidant capacity of 6.66 ± 0.19 μmol TROLOX equivalents·g^−1^ oil. This value is consistent with the antioxidant capacity of freeze-dried maqui extracts, as evaluated by Sánchez et al. [19]. It is also within the lower range of values reported for oils from various berries including blueberry (*Vaccinium corymbosum),* red raspberry (*Rubus idaeus*), marionberry (*Rubus hybrid*), and boysenberry (*Rubus hybrid*) [28].

In this study, the administration of MO has demonstrated potential health benefits by preventing liver fat infiltration into the liver of mice subjected to an HFD. The results indicate that mice fed with CD had lower total weight, lower liver weight, lower visceral fat, and lower epididymal fat when compared to mice fed an HFD. When compared to mice that received the HFD plus SO, mice corresponding to the HFD plus MO showed that MO supplementation reduced the total weight (*p* < 0.05) (i.e., equivalent to a decrease of 6%), the liver weight (*p* < 0.001) (i.e., equivalent to a decrease of 20.2%), and the visceral fat (*p* < 0.01) (i.e., equivalent to a decrease of 26.2%). These results are aligned with Juretić et al. (2021), where the effects of 12 weeks of α- and γ-tocopherol supplementation to HFD mice was measured; as a result, α- and γ-tocopherol supplementation decreased (*p* < 0.05) the body weight (by 19%) and the adipose tissue weight (by 52%), when compared to mice fed with HFD [29]. In a separate study [30], HFD-fed mice supplemented for 12 weeks with rosa mosqueta oil containing α- and γ-tocopherol showed an 18.5% reduction in body weight gain (*p* < 0.05) and a 47.4% reduction in visceral fat (*p* < 0.05) compared to HFD-fed mice supplemented with tocopherol-depleted rosa mosqueta oil [30].

HFD plus MO did not have a preventive effect on the weight gain of epididymal fat when compared to mice on the HFD plus SO, suggesting that the MO supplementation is more effective in preventing an increase in visceral fat accumulation than in that of epididymal fat. Visceral fat is known to secrete a range of pro-inflammatory cytokines, which can contribute to systemic oxidative stress, inflammation, and insulin resistance [31,32]. Notably, higher levels of HDL-cholesterol were found in the serum of HFD-fed mice supplemented with MO compared to mice receiving HFD plus SO. Tocopherols, once absorbed in the small intestine, are incorporated into chylomicrons and transferred to VLDL, LDL, and even HDL [33]; such mechanism may provide the stimulus for HDL synthesis, since an increase in HDL serum levels in patients treated with α-tocopherol (600 mg·day^−1^) has been reported [34]. Kato et al. [35] proved the effects of tocotrienols (T3s) in C57BL/6 mice fed with an HFD for 13 weeks. Body weight and adipocyte tissue (epididymal and perirenal fat) TG, low-density lipoprotein (LDL), high-density lipoprotein (HDL), total cholesterol (T-CHO), free cholesterol (F-CHO), and esterified cholesterol (E-CHO), were measured, among other parameters [35]. After 13 weeks of treatment with HFD, body weight and epididymal and perirenal fat were significantly increased compared to the control diet group (CD), and co-treatment of HFD with T3s (HFD+T3s) significantly inhibited body weight gain and perirenal fat compared to the HFD-treated group [35]. With respect to serum lipids, LDL, T-CHO, F-CHO, and E-CHO levels of the HFD+T3s group were improved compared to those of the HFD group, except for HDL levels, which were not significantly different in the presence of T3s [35].

Notably, administration of MO did not prevent an increase in glycemia and insulin levels in HFD-fed mice. Supplementation with MO reduced HOMA-IR by 27.9% compared to the HFD group supplemented with SO, although this difference was not statistically significant. Similarly, Tapia et al. [30] reported that HFD-fed mice supplemented with rosa mosqueta oil showed a 34% reduction in HOMA-IR compared to the HFD group, also without reaching statistical significance. This finding could be attributed to the specific bioactive compounds found in MO, which may target lipid pathways more effectively than glucose homeostasis pathways. It is possible that longer supplementation regimens could improve glucose homeostasis in this murine model.

MO supplementation approximately halves serum GPT concentrations, suggesting a protective effect against hepatocellular damage associated with MASLD. These findings are consistent with the study by Chung et al. [36], who evaluated the effects of α- and γ-tocopherol supplementation in an obese mouse model of non-alcoholic steatohepatitis exposed to lipopolysaccharide-induced oxidative stress [36]. The study demonstrated that both α- and γ-tocopherol provided significant protection to the liver against LPS-induced damage, reducing serum GPT concentrations by 31–34% [36]. Regarding the degree of steatosis, MO was able to reduce the degree of steatosis induced by the HFD (MO+HFD), compared to the mice group receiving the HFD+SO. Tzanetakou et al. [37] have also shown similar results in histopathology and the steatosis score in HFD+cholesterol rats supplemented with water-soluble vitamin E, where HFD+cholesterol+vitamin E rats showed mild steatosis (score of 1) and HFD+cholesterol rats showed severe steatosis (score of 3); such results proved that vitamin E administration with an HFD may prevent the development of MASLD [37].

While the present study highlights the potential of MO to mitigate MASLD in an HFD murine model, some limitations must be acknowledged. The findings from these types of models may not fully be translated to humans due to interspecies differences in hepatic and systemic metabolism. Additionally, the 12-week supplementation period, while revealing notable effects, may not reflect long-term outcomes. Optimal dosing and bioavailability (therapeutic potential) in humans remain to be established in further studies, as well as the full spectrum of the impact of MO on systemic oxidative stress and inflammation, which were not assessed here.

## 5. Conclusions

In this study, we analyzed the FA profile, tocopherol and tocotrienol content, and antioxidant capacity of MO and assessed its effects on total body and organ weights, serum markers, and liver fat infiltration in HFD-fed mice supplemented with MO for 12 weeks. MO supplementation significantly reduced (*p* < 0.05) visceral fat, GPT levels, and liver fat infiltration in obese mice on an HFD+MO compared to those on an HFD+SO. These findings suggest that MO may help in preventing metabolic alterations and liver damage associated with MASLD. Consequently, MO shows potential as an effective supplement in preventing obesity-associated fatty liver disease.

In summary, and to the best of our knowledge, the current work is the first report assessing the potential of MO in preventing liver damage induced by obesity caused by a HFD in a murine model. Therefore, its potential as a dietary supplement to prevent metabolic and liver disorders in HFD mice models ought to be studied in future research, including the assessment of the optimal dosage and duration of treatment and the potential side effects, before the exploration of its applicability in humans.

## Figures and Tables

**Table 1:** Fatty acid (FA) profile of cold-pressed maqui seed oil (MO) (g FAs·100 g^−1^ total FAs).

Systematic Name	Abbreviated Name	Content *
Myristic acid	C14:0	0.03 ± 0.04
Palmitic acid	C16:0	5.59 ± 0.06
Palmitoleic acid	C16:1 9c	0.08 ± 0.00
Stearic acid	C18:0	3.29 ± 0.01
Oleic acid	C18:1 9c	32.31 ± 0.04
Cis-Vaccenic acid	C18:1 7c	0.75 ± 0.00
Linoleaidic acid	C18:2 9t 12t	0.11 ± 0.00
Linoleic acid Nonadecenoic acid Arachidic acid	C18:2 9c 12c C19:1 7c C20:0	46.41 ± 0.05 0.09 ± 0.03 0.20 ± 0.00
α-Linolenic acid	C 18:3 9c 12c 15c	10.83 ± 0.03
Behenoic acid	C 22:0	0.31 ± 0.01
Total saturated fatty acids (TSFAs)		9.42
Total monounsaturated fatty acids (TMUFAs)		33.23
Total polyunsaturated fatty acids (TPUFAs)		57.35
Total fatty acids n-3 (TFAs n-3)		10.83

* Values are mean ± standard deviation (SD) (*n* = 3).

**Table 2:** Tocopherol concentration of cold-pressed maqui seed oil (MO) *.

MO Concentration (mg·kg^−1^ Oil)				
α-Tocopherol	α-Tocotrienol	β-Tocopherol	γ-Tocopherol	δ-Tocopherol
339.09 ± 5.15	Traces	Traces	135.52 ± 3.50	Traces

* Values are mean ± standard deviation (SD) (*n* = 2). Traces: lower content than 5 mg·kg^−1^ oil.

**Table 3:** Hydrophilic oxygen radical absorbance capacity (H-ORAC_FL_) of MO *.

Sample	H-ORAC_FL_ (μmol TROLOX Equivalents·g^−1^ Oil)
MO	6.66 ± 0.19

* Values are mean ± standard deviation (SD) (*n* = 3).

**Table 4:** Effects of cold-pressed maqui seed oil (MO) on serum biomarkers from mice *.

Biomarker	CD+SO	CD+MO	HFD+SO	HFD+MO
Fasting glucose (mg·dL^−1^)	180.6 ± 11.6 ^ac^	175.0 ± 3.7 ^ab^	205.5 ± 10.2b ^bd^	200.8 ± 8.2 ^cd^
iGTT (AUC·g^−1^)	749.6 ± 36.1 ^a^	818.8 ± 35.7 ^a^	735.3 ± 37.9 ^a^	839.2 ± 64.0 ^a^
Insulin (µg·L^−1^)	1.19 ± 0.15 ^a^	1.03 ± 0.27 ^a^	1.48 ± 0.15 ^a^	1.10 ± 0.10 ^a^
HOMA-IR	13.1 ± 3.8 ^a^	11.2 ± 7.2 ^a^	19.0 ± 5.7 ^a^	13.7 ± 2.2 ^a^
GPT (UI·L^−1^)	30.0 ± 2.7 ^a^	26.7 ± 4.7 ^b^	71.8 ± 5.0 ^b^	34.2 ± 2.6 ^c^
GOT (UI·L^−1^)	70.3 ± 8.7 ^a^	58.3 ± 5.7 ^a^	83.7 ± 7.6 ^a^	59.7 ± 6.8 ^a^
TG (mg·dL^−1^)	49.0 ± 2.8 ^a^	62.0 ± 4.2 ^a^	53.5 ± 5.7 ^a^	62.7 ± 4.3 ^a^
T-Chol (mg·dL^−1^)	172.3 ± 5.7 ^a^	159.4 ± 2.9 ^ab^	185.7 ± 5.7 ^bc^	187.6 ± 3.2 ^c^
HDL-Chol (mg·dL^−1^)	103.3 ± 7.8 ^a^	110.9 ± 4.5 ^ab^	116.4 ± 5.0 ^bc^	130.9 ± 3.8 ^c^

* Biochemical parameters from mice supplemented with cold-pressed maqui seed oil. Serum was obtained from peripheral blood and used to measure lipid and liver biochemical profiles. Intraperitoneal glucose tolerance test (iGTT), performed after 4 h fasting is represented by the area under the curve (AUC). HOMA-IR, homeostatic model assessment for insulin resistance. Abbreviations: GPT (hepatic glutamate pyruvate transaminase), GOT (hepatic glutamate oxaloacetate transaminase), T-Chol (total cholesterol), HDL-Chol (HDL-cholesterol), CD (control diet), HFD (high-fat diet), SO (sunflower oil), and MO (cold-pressed maqui seed oil). Data are presented as the mean ± standard error of the mean (SEM). Statistical differences were determined using ordinary two-way ANOVA test, followed by Tukey’s comparative test. In each column, values with different superscript letters (^a–d^) indicate significant differences (*p* < 0.05); *n* = 6 animals for each group.

**Table 5:** Fatty liver infiltration through histological analysis. Steatosis mice score *.

Histological Feature	CD+SO	CD+MO	HFD+SO	HFD+MO
Steatosis				
Macrovesicular	1	1	2	1
Microvesicular	0	1	3	2
Hypertrophy	0	0	1	0
Inflammation				
Number of inflammatory foci/field	0	0	0	0
Total score	1/12 ^a^	2/12 ^a^	6/12 ^b^	3/12 ^a^

* Fatty liver infiltration was evaluated through histological analysis in mice fed with different diets, including control diet with sunflower oil (CD+SO), control diet with cold-pressed maqui seed oil (CD+MO), high-fat diet with sunflower oil (HFD+SO), and high-fat diet with cold-pressed maqui seed oil (HFD+MO). Steatosis was assessed by scoring macrovesicular and microvesicular fat, hypertrophy, and inflammation foci. Statistical analysis was performed using ordinary two-way ANOVA test, followed by Tukey’s comparative test to determine significant differences between groups. In each column, values with different superscript letters (^a, b^) indicate significant differences (*p* < 0.05); *n* = 6 animals for each group. * Based on Liang et al. [18].

## Data Availability

All the data are contained within the manuscript.
